# Increase in Ultrasound-guided Nerve Blocks Observed After Implementation of a Nerve Block Supply Cart in the Emergency Department

**DOI:** 10.5811/westjem.53900

**Published:** 2026-07-16

**Authors:** Aurora Jin, Allison Cohen, Aileen Virella, Nicholas Bielawa, Martin Miller, Mathew Nelson

**Affiliations:** * North Shore University Hospital, Department of Emergency Medicine, Manhasset, New York; † Plainview Hospital, Department of Emergency Medicine, Plainview, New York; ‡ North Shore University Hospital, Department of Anesthesiology, Manhasset, New York

## Abstract

**Introduction:**

Ultrasound-guided nerve blocks (UGNB) are an effective and safe method of pain control that are increasingly used for pain management in the emergency department (ED). However, performing these procedures is often time-intensive due to the need to locate and gather necessary supplies. Our ED recently implemented use of a nerve block cart, which contains all the supplies needed for performing a nerve block. Our objective in this quality improvement study was to assess whether the presence of an ED nerve block supply cart was associated with an increase in the number of UGNBs performed.

**Methods:**

We conducted a retrospective review of point-of-care ultrasound examinations performed in a single academic quaternary-care community ED over 22 months, looking at 11-month time periods before and after implementation of an ED nerve block supply cart in March 2023. Our primary outcome measure was documentation of a UGNB, and our secondary outcome measure was the type of UGNB performed.

**Results:**

We reviewed 14,321 ultrasound exams over 22 months—11 months pre-cart (6,853) and 11 months post-cart (7,468) implementation. We noted a statistically significant increase in the number of UGNBs, from 21 performed in the pre-cart period (0.31% of all ultrasound exams [95% CI, 0.17–0.43%], to 50 UGNBs in the post-cart period (0.67% of all ultrasound exams [95% CI, 0.46–0.86%], *P* < .01). The fascia iliaca block was the most common UGNB performed in both the pre- (52.4%) and post-cart (62.0%) periods.

**Conclusion:**

We found that the deployment of an ED nerve block supply cart was associated with an increased number of ultrasound-guided nerve blocks performed by emergency clinicians in our hospital.

## INTRODUCTION

Ultrasound-guided nerve blocks (UGNB) are an effective method of pain control, and they are increasingly becoming a core component of multimodal pain management in the emergency department (ED).^1^,^2^ A survey of academic EDs with ultrasound fellowships found a 16% increase in the number of EDs that reported performing UGNBs from 2016 to 2022.^1^,^2^ A literature review also demonstrated an increasing number of academic publications discussing UGNBs between 2003–2025, with two-thirds of all articles published since 2020.^3^ Effective procedural setup is key to performing UGNBs, and the availability of specialty supplies, such as nerve block needles, anesthetic, and intralipid, improves the success of ED nerve block programs.^4^,^5^ To date, no emergency medicine literature has documented whether efforts to improve procedural setup and access to supplies increases the number of UGNBs performed in the ED.

In March 2023, our ED and anesthesiology service jointly implemented a quality improvement (QI) project to facilitate emergency clinician performance of UGNBs; the QI project entailed the creation of an ED nerve block cart, which holds all the supplies needed to perform a UGNB. A standard medical supply cart with six drawers includes long- and short-acting anesthetics, nerve block needles, intralipid, syringes and regular needles, sterile ultrasound gel, and sterile gloves. (For exact supplies, see Table 1.) The cart is stationed in a central zone of the ED and is jointly stocked by the anesthesiology department. The information on charges for cart supplies was provided by our ED supply distribution operations coordinator and the anesthesiologist who handles stocking of the anesthetics. Cart contents were determined based on hospital anesthesiology policy and emergency clinician recommendations regarding the size of block needles. The primary objective of this QI project was to determine whether having an ED nerve block cart was associated with an increased number of UGNBs performed.

## METHODS

We conducted a retrospective quality review of all point-of-care ultrasound (POCUS) exams performed in a single, quaternary-care, academic-affiliated ED from April 1, 2022–January 31, 2024. We referenced the SQUIRE 2.0 guidelines to ensure that our study meets established standards for QI research.^6^ The study was conducted with the assistance of our emergency ultrasound division. We chose to review POCUS imaging to identify UGNBs performed and interpret this number in the context of all scans performed in our ED, which has many ultrasound-trained clinicians. Per departmental policy, all UGNBs must be performed by a credentialed clinician, images and/or clips must be saved, and a procedure note must be documented in the electronic health record (EHR) noting the type and amount of anesthetic used. Thus, all UGNBs were performed either by credentialed clinicians, which included the study authors, or directly under their supervision.

We further describe below elements of optimal retrospective chart review that we adhered to, as delineated in Worster and Bledsoe 2005.^7^ Our sampling method was as follows: We reviewed all POCUS exams performed in the ED for UGNBs—our dependent variable—over 22 months, representing 11 months prior to (pre-cart) and 11 months after (post-cart) nerve block cart implementation—our independent variable. An ultrasound fellow and a senior ultrasound fellowship-trained attending reviewed the scans using the QPathE software platform, which archives ED POCUS exams. (A small number were reviewed by another ultrasound fellow and a third-year emergency medicine resident with additional experience in preparation for ultrasound fellowship.) All abstractors had sufficient prior training and clinical experience in recognizing a nerve block on ultrasound; thus, abstractor performance was not explicitly monitored. Chart review was performed on our EHR system, Sunrise, for all UGNBs visualized on QPathE and included in the study.

**Population Health Research Capsule**

What do we already know about this issue?

*Ultrasound-guided nerve blocks (UGNB) are an effective and safe method of pain control, but many factors may hinder their use in the emergency department (ED)*.

What was the research question?

*Was implementation of an ED nerve block supply cart associated with an increase in the number of UGNBs performed?*

What was the major finding of the study?

*The number of UGNBs increased from 0.31% (95% CI, 0.17–0.43%) to 0.67% (95% CI, 0.46–0.86%) of all ultrasound exams after cart deployment (P < .01)*.

How does this improve population health?

*Implementing nerve block carts in the ED may encourage the use of UGNBs to control pain and could decrease rates of complication for many acute conditions*.

Abstractors were also proficient with use of the EHR to identify nerve block documentation, as this process is identical to how charts are abstracted for weekly ED POCUS quality review. Institutional review board review and exemption were determined to be not required per institutional review and health system guidelines regarding QI projects. Additionally, our department reviews 100% of all ED ultrasounds for quality assurance.

All sonography exams were reviewed for the presence of UGNBs. Our case selection criteria were as follows: We included scans if images and/or clips of a UGNB were identified. Included scans had to show an echogenic needle approaching a hyperechoic nerve bundle, followed by instillation of anesthetic around the nerve bundle. The UGNB was then confirmed through chart review of the procedure note on the EHR to ensure that the scan was identified accurately. The type of UGNB was also recorded. To manage any missing data, we excluded scans if patient and/or confirmatory procedure note information were absent or incorrect. The data were transcribed in a standardized abstraction form in Microsoft Excel. We analyzed the data using a chi-squared test to compare the percentages of UGNBs performed out of all ultrasound exams identified during the pre- and post-cart time periods.

## RESULTS

We reviewed 14,321 ultrasound exams for this study (Table 2). A total of 6,853 were performed in the pre-cart period, while 7,468 were performed in the post-cart period. One scan, which was initially identified as a possible UGNB, was excluded upon chart review confirming it was an ultrasound-guided trigger point injection. No scans had to be excluded due to lack of or incorrect patient or ultrasound information. While 21 UGNBs, or 0.31% (95% CI, 0.17–0.43%), were performed in the pre-cart period, 50 UGNBs, or 0.67% (95% CI, 0.46–0.86%), were performed in the post-cart period, representing a statistically significant increase (*P* < .01). The fascia iliaca block was the most common UGNB performed in both the pre-cart (11) and post-cart (31) periods (Table 3). Using the data presented in Table 1, we estimated that the annual cost to stock the cart would be around $1,200 based on the number of UGNBs performed post-cart implementation.

## DISCUSSION

Our study demonstrated that implementation of an ED nerve block supply cart more than doubled the number of UGNBs performed in our ED. Ultrasound-guided nerve blocks are a safe and effective means of providing analgesia in the ED and are used for many indications, including fractures, intra-abdominal conditions, and facilitation of procedures.^1^,^2^,^8^–^14^ The UGNB is particularly useful for hip fractures and has been given a “strong” recommendation by the American Academy of Orthopedic Surgeons for use in preoperative pain management.^15^ Use of UGNBs for hip fractures has been found to improve patient-centered outcomes including reducing opioid use, decreasing postoperative delirium events, and improving morbidity and mortality.^9^,^16^–^17^ Thus, UGNBs are increasingly becoming the standard of care for pain management in the ED.

Improving barriers to performing UGNBs has been an ongoing challenge for emergency clinicians. There are numerous proposed limiting factors, including the time requirements to perform the procedure, available personnel, and locating the necessary supplies.^4^,^5^ A prior study demonstrated that instituting an ED nerve block team led to a rise in both the number and variety of blocks performed in the ED.^18^ Our study adds an additional potential solution to the challenge of locating supplies for performing UGNBs. Having an available, fully stocked nerve block cart in the ED would likely reduce the time needed to gather supplies as they would be located in one central location. Additionally, our study highlights the successful results of an interdepartmental collaborative initiative between the ED and anesthesiology to increase use of of UGNBs in the ED.

The general reception to the cart has been positive due to ease of use and ability to make UGNBs easier to perform. We believe that the cart’s ability to facilitate UGNB procedures clears one of the many hurdles that EDs can face in encouraging clinicians to perform more UGNBs. Additional system-level hurdles include procedure credentialing, UGNB educational initiatives, buy-in from other departments, ability to conduct quality review of the procedures performed, and supply availability and cost.^4^,^5^ The startup cost of the project, including the supplies and cart itself, was approximately $2,298, and we estimate a yearly maintenance cost of $1,200 based on cart supply information (Table 1) and our data over the one-year period after project implementation. Additional factors that would influence this estimate apart from the number of UGNBs performed include any clinician preference for specific anesthetics, nerve block needles, and/or other supplies with multiple options that vary in cost.

Further research examining the factors that can improve procedural setup and increase UGNBs performed in the ED will be extremely important as UGNBs become the standard of care for various indications. Our results demonstrate that an initial investment in setting up an ED nerve block cart may be a useful consideration in EDs looking to significantly increase the number of UGNBs performed by emergency clinicians.

## LIMITATIONS

The main limitation of this study is that it was conducted at a single center with a strong pre-existing academic ultrasound division. In many EDs, this is not the case; therefore, the barriers to performing UGNBs are likely multifaceted and not remedied by simply improving access to supplies. We suspect that, at minimum, experienced clinicians and UGNB education must already be in place to demonstrate similar results. A second limitation is that our study was a retrospective chart review. Some UGNBs may have been missed if they were not recorded, but additional non-UGNB images that were not recorded might also exist, both of which would affect our reported percentages of UGNBs performed. However, we feel this is unlikely in an ED with a strong ultrasound presence and a formal policy of recording all scans and documenting them with a procedure note.

A third limitation of this study is that chart reviewers were not blinded to the study objective when reviewing scans, and inter-rater reliability and chart accuracy review were not formally performed. However, if an UGNB was found, chart review was conducted to ensure that it was correctly identified. Given the objective nature of chart abstraction for presence or absence of a nerve block, this was thought to represent little risk of bias. Another limitation is that clinical outcome data, such as improvement of pain and reduction of opioid use, were not obtained; thus, the extent to which the UGNBs performed were successful is unknown. This study was also limited by the small sample size of UGNBs identified. As this was a pilot program to assess the efficacy of the QI project, a one-year mark was chosen as the period for review. Despite the small sample size, our results indicate the effectiveness of instituting a nerve block cart to increase the frequency of the procedure. Future studies are needed to ensure that this practice continues.

Finally, the increase in the number of UGNBs performed that we identified may be part of a nationwide trend that has been observed in EDs with ultrasound fellowships to increase multimodal pain control, especially for hip fractures.^1^,^2^ This increase may also be attributed to multiple pre-existing interventions at our hospital, including UGNB educational initiatives for emergency clinicians and support from the departments of anesthesiology and orthopedics. Because these initiatives were already in place prior to introduction of the nerve block cart, we suspect that the significant increase in UGNBs performed was observed specifically in association with supply cart implementation.

## CONCLUSION

Introduction of a nerve block cart was associated with an increased number of ultrasound-guided nerve blocks performed in our ED. Accessibility of supplies for emergency clinicians who work in a high-acuity, fast-paced environment may play a significant role in driving procedural awareness and performance. Further studies are needed to determine whether similar interventions within EDs at different institutions would have similar results.

## Supplementary Information



## Figures and Tables

**Table 1:** Contents and cost of a nerve block supply cart created as part of a quality improvement project to increase the number of ultrasound-guided nerve blocks performed in the emergency department.

Supply item	Approximate quantity	Cost per unit	Total cost
Ropivacaine 0.5% (150 mg/30 mL)	10	$3.17	$31.70
Ropivacaine 0.2% (40 mg/20 mL)	10	$2.64	$26.40
Bupivacaine with epinephrine 0.5% (150 mg/30 mL)	10	$7.23	$72.30
Bupivacaine with epinephrine 0.25% (25 mg/10 mL)	10	$6.51	$65.10
Lidocaine 2% (2 mL)	25	$1.73	$43.25
Intralipid 20% (250 mL)	1	$17.50	$17.50
Arrow UltraQuik needles 21G 100 mm with tubing	10	$9.48	$94.80
Pajunk SonoBlock II facet needles 22G 50mm with tubing	10	$12.25	$122.50
Pajunk SonoBlock II facet needles 21G 100 mm with tubing	10	$13.65	$136.50
18-gauge blunt needles	30	$0.07	$2.10
50 mL syringes	5	$0.40	$2.00
20 mL syringes	10	$0.23	$2.30
10 mL syringes	10	$0.11	$1.10
3 mL syringes	15	$0.04	$0.60
1 mL syringes	15	$0.32	$4.80
3-way stopcocks	5	$0.54	$1.62
Luer red caps	40	$0.47	$18.80
ChloraPrep sticks	15	$0.85	$12.75
Tegaderm transparent dressing, 6x8 inches	15	$1.17	$17.55
Sterile gauze sponges, 4x4 inches	15	$0.04	$0.60
Sterile ultrasound gel	15	$0.78	$11.70
Sterile gloves (multiple sizes)	80	$1.40	$112.00
All items	-	-	$797.97

**Table 2:** Total ultrasound images reviewed before and after nerve block supply cart implementation with number of ultrasound-guided nerve blocks performed respectively during each time period.

Category	Pre-cart (4/1/2022–2/28/2023)	Post-cart (3/1/2023–1/31/2024)
Total days reviewed	334	337
Total ultrasound exams	6,853	7,468
Total nerve blocks	21 (0.31%)	50 (0.67%)

**Table 3:** Types of ultrasound-guided nerve blocks performed during the time periods before and after implementation of the nerve block supply cart.

Nerve block type	Pre-cart (4/1/2022–2/28/2023)	Post-cart (3/1/2023–1/31/2024)
Fascia iliaca	11 (52.38%)	31 (62.00%)
Sciatic nerve	0 (0.00%)	5 (10.00%)
Erector spinae	1 (4.76%)	5 (10.00%)
Serratus anterior	3 (14.29%)	1 (2.00%)
Radial nerve	1 (4.76%)	2 (4.00%)
Median nerve	2 (9.52%)	3 (6.00%)
Ulnar nerve	0 (0.00%)	1 (2.00%)
Posterior tibial nerve	1 (4.76%)	1 (2.00%)
Supraclavicular	2 (9.52%)	1 (2.00%)
