# Supplementary material for: Increase in Ultrasound-guided Nerve Blocks Observed After Implementation of a Nerve Block Supply Cart in the Emergency Department
Source: West J Emerg Med. 2026 Jul 16;27(4):1034–8. doi: 10.5811/westjem.53900 (PMC13436673; doi:10.5811/westjem.53900)
Supplement: Supplementary file 1 [file wjem-27-1034-s001.pdf]

Appendix 1 – Table of Nerve Block Cart Contents and Cost

Supply Item	Approximate Quantity	Cost Per Unit	Total Cost
Ropivacaine 0.5% (150mg/30mL)	10	$3.17	$31.70
Ropivacaine 0.2% (40mg/20mL)	10	$2.64	$26.40
Bupivacaine with epinephrine 0.5% (150mg/30mL)	10	$7.23	$72.30
Bupivacaine with epinephrine 0.25% (25mg/10mL)	10	$6.51	$65.10
Lidocaine 2% (2mL)	25	$1.73	$43.25
Intralipid 20% (250mL)	1	$17.50	$17.50
Arrow UltraQuik needles 21G 100mm with tubing	10	$9.48	$94.80
Pajunk SonoBlock II Facet needles 22G 50mm with tubing	10	$12.25	$122.50
Pajunk SonoBlock II Facet needles 21G 100mm with tubing	10	$13.65	$136.50
18-gauge blunt needles	30	$0.07	$2.10
50mL syringes	5	$0.40	$2.00
20mL syringes	10	$0.23	$2.30
10mL syringes	10	$0.11	$1.10
3mL syringes	15	$0.04	$0.60
1mL syringes	15	$0.32	$4.80
3-way stopcocks	5	$0.54	$1.62
Luer red caps	40	$0.47	$18.80
ChloraPrep sticks	15	$0.85	$12.75
Large Tegaderms 6x8in	15	$1.17	$17.55
Sterile gauze sponges 4x4in	15	$0.04	$0.60
Sterile ultrasound gel	15	$0.78	$11.70
Sterile gloves (multiple sizes)	80	$1.40	$112.00
**All items**	**-**	**-**	**$797.97**
